# Enhancing urban blue-green landscape quality assessment through hybrid genetic algorithm-back propagation (GA-BP) neural network approach: a case study in Fucheng, China

**DOI:** 10.1007/s10661-024-12558-6

**Published:** 2024-04-04

**Authors:** Ding Fan, Nor Zarifah Binti Maliki, Siwei Yu, Fengcheng Jin, Xinyan Han

**Affiliations:** 1 School of Housing, Building, and Planning, Universiti Sains Malaysia, George, Penang 11800 Malaysia; 2 School of Art and Design, Leshan Normal University, Leshan, 614000 Sichuan Province China

**Keywords:** Blue-green space, Landscape quality, Ecological effects, Prediction and optimization, GA-BP

## Abstract

This study employs an artificial neural network optimization algorithm, enhanced with a Genetic Algorithm-Back Propagation (GA-BP) network, to assess the service quality of urban water bodies and green spaces, aiming to promote healthy urban environments. From an initial set of 95 variables, 29 key variables were selected, including 17 input variables, such as water and green space area, population size, and urbanization rate, six hidden layer neurons, such as patch number, patch density, and average patch size, and one output variable for the comprehensive value of blue-green landscape quality. The results indicate that the GA-BP network achieves an average relative error of 0.94772%, which is superior to the 1.5988% of the traditional BP network. Moreover, it boasts a prediction accuracy of 90% for the comprehensive value of landscape quality from 2015 to 2022, significantly outperforming the BP network’s approximate 70% accuracy. This method enhances the accuracy of landscape quality assessment but also aids in identifying crucial factors influencing quality. It provides scientific and objective guidance for future urban landscape structure and layout, contributing to high-quality urban development and the creation of exemplary living areas.

## Introduction

Rapid urbanization poses a variety of challenges, including those related to social sustainability, climate resilience, and land-use intensification. In response, national policies require urban planning and construction to consider the impact on nature and maintain a harmonious relationship between humans and the environment. To address these challenges, the blue-green landscape system offers an integrated solution (Leggett & Carter, 2019), which creates an interconnected system of water and green spaces in cities to reduce the pressure on energy, water, and climate; enhance the quality of life; and achieve key concepts of energy efficiency, livability, and sustainability (Wang et al., 2020a, 2020b; Zhang et al., 2021a, 2021b). Water bodies and vegetation landscapes are critical components of urban blue-green systems and play essential roles in assessing urban ecological and environmental quality (Macsimovici, 2019; Mu et al., 2020). They also serve as sources of information that shape users’ perceptions of landscape quality and act as natural media and material carriers to maintain a harmonious relationship between humans and nature (Bera et al., 2022). The scientific and rational planning of blue-green landscapes is crucial for assessing the quality of urban environmental landscapes (Cheng & Wu, 2020; Li & Lange, 2023). It enhances a city’s image and is vital in providing a healthy living space for human beings and improving the urban ecological environment (Lei & Jain, 2022).

Scientific planning of blue-green landscape systems is crucial for enhancing urban landscape quality and ensuring healthy living spaces. An urban landscape system is complex, non-linear, and requires real-time evaluation (Nicolini, 2022). A comprehensive evaluation of landscape quality must consider urban social and economic development indicators. Artificial neural networks (ANNs) are effective tools for handling uncertainty and are suitable for prediction, optimization, and evaluation (Jahani et al., 2022; Zou et al., 2021). However, traditional Backpropagation (BP) neural networks have limitations that can be addressed by optimizing the network using a Genetic Algorithm (GA). A mixed training algorithm that combines the strengths of both GA and neural networks can optimize the network and achieve better calculation results (Jiao et al., 2023). The proposed comprehensive evaluation method for urban landscape quality aims to provide a scientific basis for urban landscape planning and design, thereby promoting the sustainable development of urban ecology and human society.

The assessment of ecological service quality in landscape systems is a complex and multifaceted undertaking that encompasses resource, social, and economic objectives, levels, and factors (Ke, 1992). Various methods have been applied for the comprehensive evaluation of multiple indicators, such as factor analysis (Bera et al., 2022), the Delphi method (Zheng et al., 2017), and hierarchical analysis (Wu et al., 2020a, 2020b). However, these mathematical models possess certain shortcomings and lack a theoretical basis for weight determination, rendering them impractical for real-world applications. Constructing landscape structures for water bodies and green areas involves a non-linear mapping process that encompasses a complex interplay of various natural and anthropogenic disturbance factors and the spatial characteristics of the landscape. In contrast, BP neural networks offer a theoretical and computational intelligence-based approach for modeling and simulating different layers of the landscape system. Incorporating momentum factors into the optimization process enhances the usability of the optimization algorithm, making it more precise and efficient than the standard BP networks (Xu et al., 2016). Jahani et al. (2022) emphasized the importance of considering the reliability of indicators, weights, and data in the BP neural network-based ecological evaluation method for urban public green spaces to improve the scientific and accurate assessment of green spaces, thereby demonstrating the effectiveness of the BP neural network algorithm in the ecological evaluation of urban public green spaces. The Genetic Algorithm-Back Propagation neural network has demonstrated superior global convergence and stability, making it a valuable tool for accurately assessing landscape quality levels (Nikbakht et al., 2021). Recent studies have confirmed the effectiveness of GA-BP neural networks for landscape evaluation. For instance, Wu et al., (2020a, 2020b) utilized a GA-BP neural network to assess land ecological quality in Yuxi City from 2001 to 2015, and the GA-optimized model was found to exhibit faster convergence, higher accuracy, and less error during training and prediction than traditional BP neural networks (Pu & Cai, 2022). In another study, Nikbakht et al. (2021) optimized hidden layers, integration points, and the number of neurons per layer using the GA algorithm, leading to a significant improvement in the prediction accuracy of the neural network for the stress distribution of structures. Yang, 2020 employed the BP optimization algorithm to measure waterfront and plant landscape structures on a small scale, and their results demonstrated that the algorithm’s convergence speed and warp were stronger than those of other algorithms, leading to a more accurate assessment of landscape quality levels (Yang, 2020). Finally, Bera et al. (2022) used a GA-BP neural network to develop an evaluation model for urban green development, which successfully trained and tested 20 valid indicators, such as sewage treatment, green coverage, forest coverage, urbanization level, and population size, resulting in data clustering analysis and prediction results consistent with objective reality. In conclusion, GA-BP neural networks are powerful tools for assessing landscape quality and evaluating ecological service quality in landscape systems, providing accurate and efficient solutions to practical problems (Pu & Cai, 2022).

As discussed above, the traditional BP neural network has limitations that can be solved by optimizing the network using a genetic algorithm (GA). The hybrid training algorithm combines the advantages of a genetic algorithm and a neural network and can optimize the network and obtain better computational results. It can be observed from the literature that the multi-index comprehensive evaluation method based on mathematical models has some defects and is not practical. Recent studies have confirmed the effectiveness of genetic neural networks in landscape evaluation, such as Wu et al., who found the advantages of genetic neural network models in training and prediction and obtained data analysis and prediction results that conform to objective reality. This is a meaningful exploration.

However, we found that there is a lack of empirical studies on applying the GA-BP neural network model to evaluate the quality of the urban blue-green landscape (UBL) and predict its future changes, and no clear guidance on how to select and weigh the relevant indicators for this model. Therefore, this study aims to explore how to use the GA-BP neural network model to effectively evaluate and predict the quality of UBL based on multiple indicators and analyze its implications for urban planning and design. UBL is an important component of the urban ecological environment and is of great significance for improving the quality of life and promoting sustainable urban development (Song et al., 2022). Evaluating the quality of UBL is a complex nonlinear problem that requires consideration of multiple influencing factors, such as socio-economic development, ecological environment conditions, and landscape pattern characteristics (Jiao et al., 2023; Sun et al., 2017a, 2017b). The GA-BP neural network model can handle large amounts of data and pattern recognition and improve the accuracy and efficiency of evaluation (Hua et al., 2020). This method helps to evaluate the comprehensive value and level of landscape quality more objectively and reconsiders the rationality, scientific, and sustainability of the UBL structure. This study contributes to better landscape planning and design in the future.

## Study area and methods

### Study area

**Fig. 1:**
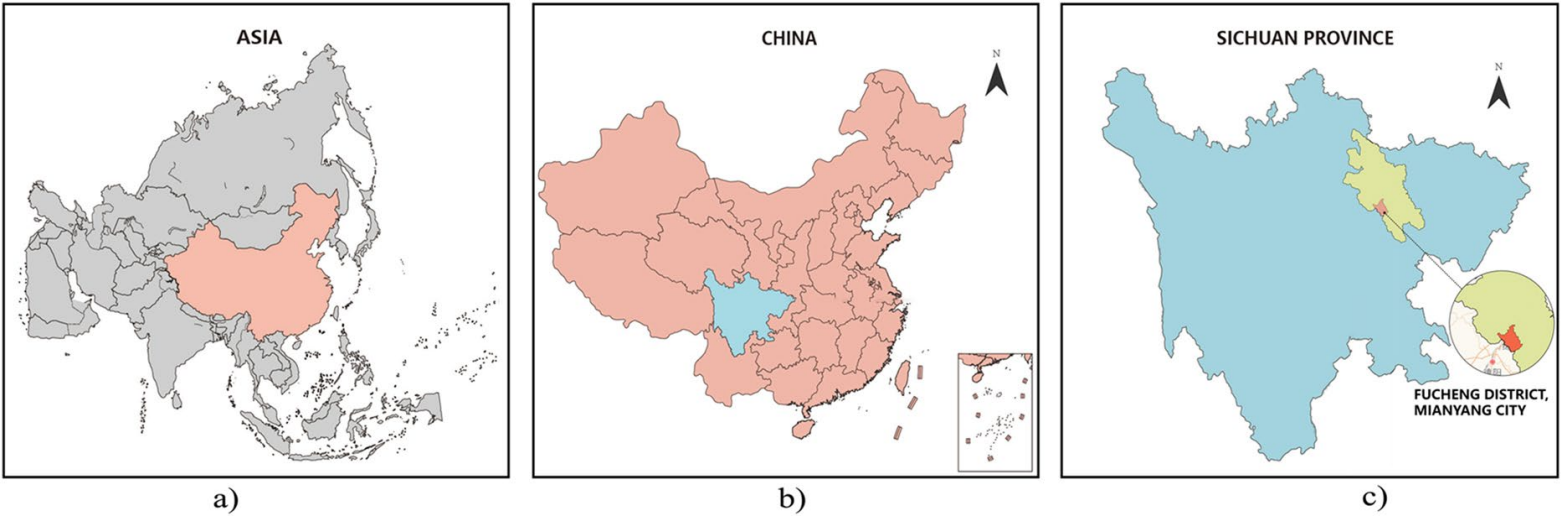
Study area location (self-drawing)

Fucheng District (Fig. 1a–c) is situated on the west bank of the Fu River to the west of Mianyang City. It covers a total area of 55.47 km^2^, with an average elevation of 551 m and an average annual temperature of 16.3 °C. According to the seventh national census, the population is 1.31 million. With the opportunities presented by the Chengdu-Chongqing dual-city economic circle, Fucheng District has made significant progress in urban construction and development, achieving a GDP growth rate of approximately 7% in the past 2 years. It is expected that the urbanization rate will reach 82% by 2022. In terms of the urban environment, the city boasts a forest area of 12.3 thousand hm^2^, with a vegetation coverage rate of 22.21%, 12 comprehensive parks, and a per capita green space area of 10.39 m^2^. The water area and water facilities cover an area of 0.4804 thousand hm^2^, and the per capita available water resources amount to 1090 m^3^ (Statistical Yearbook Platform). Infrastructure and public service facilities are well equipped, and the city has been recognized as a United Nations model city for improving the living environment.

### Research method

#### Data type and sources

According to the requirements of the construction of the landscape quality evaluation index system and output validity, three types of data were selected: socio-economic data (such as population, economic output, employment), ecological environment data (such as climate, water environment, vegetation coverage), and landscape pattern characterization data (such as the distribution, morphology, and scale of the water body and green space), which mainly come from local government statistical data, socio-economic surveys, environmental protection departments, satellite remote sensing data, etc. (as seen in Table 1). These data served as support for the research content.

**Table 1:** Research data type and sources (2020–2023)

Data type	Data sources
Statistical data	Statistical Yearbooks Sharing Platform (https://www.yearbookchina.com)
Government annual reports	Official Website of Fucheng District Government, Mianyang City, Sichuan Province, China (http://www.myfc.gov.cn/)
Spatial data	National Integrated Earth Observation Data Sharing Platform (http://www.chinageoss.cn/)
Meteorological and hydrological data	National Meteorological Science Data Centre (http://www.cma.gov.cn)

#### Research path

In this study, we quantitatively analyzed the quality of the blue-green landscape in Fucheng District from multiple levels and then used the GA-BP neural network model to predict and test the accuracy of the assessment level, so that we can intuitively determine the key factors affecting the quality of the landscape. There are main steps to this process: landscape information extraction, landscape index analysis, evaluation index system construction, assessment criteria, and level, GA-BP neural network modeling, and model validation, specifics are as follows:

- 1. Landscape information extraction

**Fig. 2:**
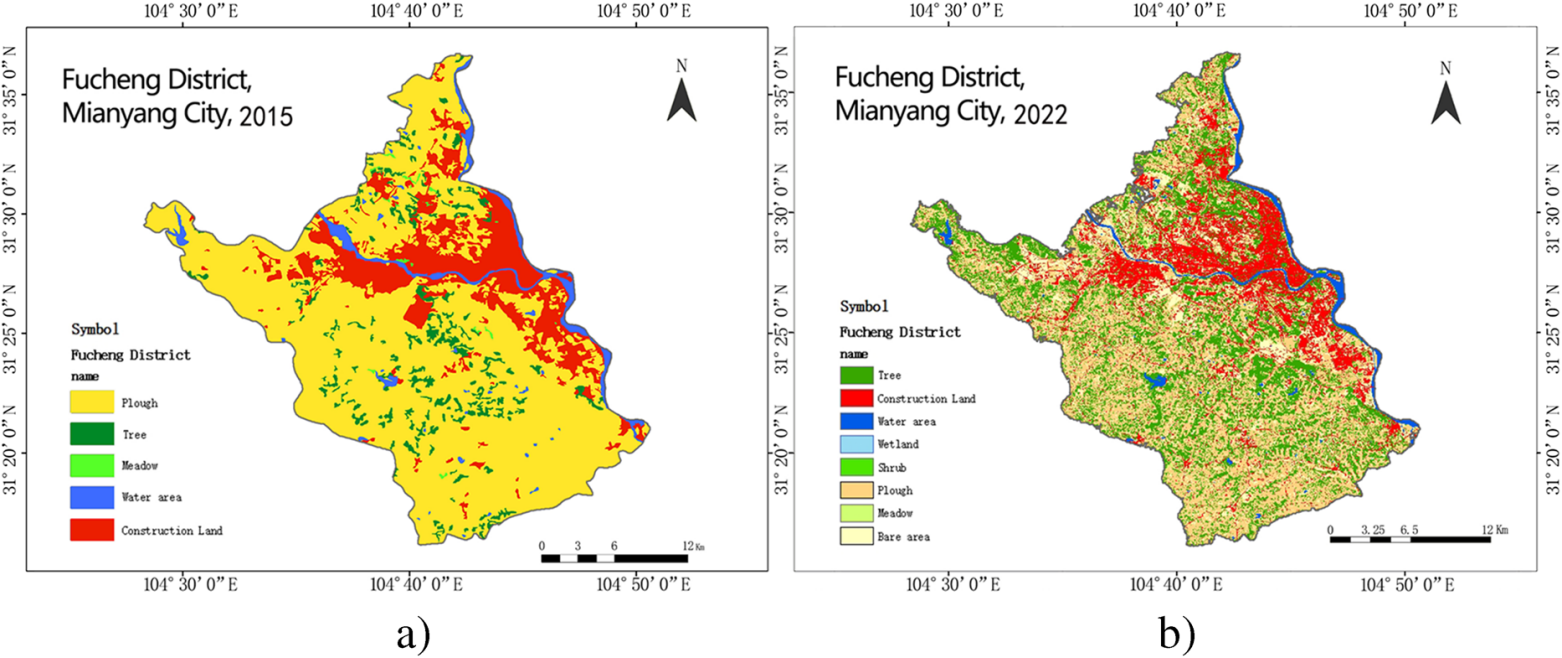
Land use comparison of Fucheng District, 2015 and 2022 (self-drawing)

**Fig. 3:**
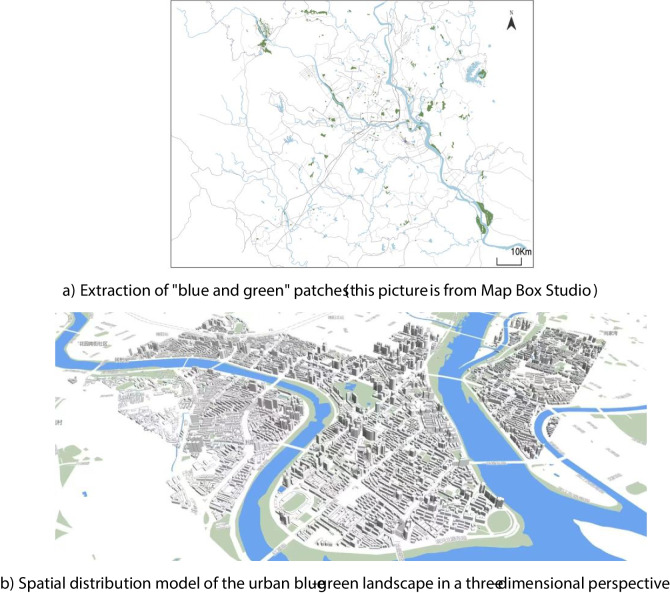
Legend of the distribution of blue and green landscapes in the study area (the above figures are self-drawing)

We used satellite images and GIS (Geographic Information System) technology to obtain the urban blue-green landscape information of Fucheng District from 2015 to 2022 (as shown in Fig. 2a, b). These data were obtained from the GF-1 satellite impacts provided by the China Resources Satellite Application Center (CRESDA), with a resolution of 15 m. We used ArcGIS software to preprocess the data, including steps such as image alignment, radiometric correction, and atmospheric correction. We then used the MAPBOX studio to extract information about water bodies and green space patches and summarize the characteristics of landscape elements, such as area, shape, and distribution (as shown in Fig. 3).

As shown in Fig. 2a, b, using Landsat TM/ETM/OLI remote sensing images as the main data source, the urban land use of the Fucheng district at the beginning of the study period in 2015 and the end of the study period in 2022 is shown using a geographic information system (GIS). As shown in Fig. 3, the map box map analysis tool was used to show the distribution and three-dimensional perspective of the blue and green patches, which more clearly and intuitively reflect the current status of the spatial organization pattern of the Fucheng District.

From Fig. 3, it can be seen that the blue and green landscape spaces in Fucheng District show regularity and diversity. The water body patches are mainly distributed along the Fuling River, forming a blue corridor that provides the city with ecological nourishment and landscape beautification. The green patches are mainly concentrated in the city center and periphery, forming several green islands and providing the city with recreation and air purification functions. Water bodies and green patches are intertwined with each other, forming a composite blue-green landscape structure that provides diversified ecological services and landscape benefits for the city (Wang et al., 2020a, 2020b).

The analyses in Figs. 2 and 3 play an important role in later landscape quality assessments. On one hand, it provides the basic information and characteristics of the blue-green landscape space in the study area; on the other hand, it also provides data support for selecting and calculating the variables affecting the landscape quality (Wei et al., 2022), establishing and training the neural network model, which provides a method basis for evaluating and predicting the landscape quality, and also provides a guide to make suggestions for improving the landscape quality at a later stage.

- 2. Landscape index analysis

We used a variety of landscape indices to analyze the spatial distribution, pattern, and size characteristics of water bodies and green patches in the study area (Zhang et al., 2019). Therefore, we used ArcGIS software to categorize the satellite images and extract information on water bodies and green space patches. Fragstats software was used to calculate the individual landscape index. For example, the number of patches of water bodies and green spaces in the study area (NP), the number of patches of water bodies and green spaces per square kilometer (PD), the average of the patch areas of water bodies and green spaces (AREA_MN), and the spatial correlation between patches of the same category (AI).

The landscape index is a numerical index used to quantify and describe the spatial structure, form, and function of the landscape and can reflect the diversity, complexity, connectivity, stability, and other characteristics of the landscape (Bian et al., 2023). Based on these landscape indices, we can better understand the spatial characteristics of blue-green landscapes in the study area, thereby providing an important basis for subsequent assessment and prediction (Cong et al., 2019).

- 3. Construction of evaluation index system

First, the principles of indicator extraction must be clarified. The indicator system can comprehensively reflect the structure, functions, benefits, and trends of the evaluation object, provide an objective and comprehensive basis for evaluation, and simultaneously help to identify the key factors affecting the evaluation object to provide scientific and reasonable guidance for future planning and design (Wang et al., 2021; He et al., 2020). Therefore, based on the principles of science, context, sustainability, and objectivity, we extracted indicators that can reflect the quality of urban blue-green landscapes from multiple perspectives as follows:

On the one hand, scientific and context: The indicator set should be representative, relevant, and scientifically reflect the core elements of the evaluation object. Based on data from the Fucheng District Statistical Yearbook 2015–2022 (Statistical Yearbook Platform, 2023), relevant factors affecting the quality of the landscape were identified and analyzed. On the other hand, the principle of Sustainable Development: The establishment of an indicator system should reflect Fucheng District’s ecological wisdom in terms of the natural landscape, humanistic landscape, and resource protection and utilization, ensuring a sustainable approach (Cecchini et al., 2019). Furthermore, the principle of objectivity: With the assistance of satellite maps and GIS, the landscape information of the city’s blue-green spaces was extracted, and the landscape scale, pattern, and distribution characteristics were analyzed using landscape indices, providing objective and accurate data for the evaluation process (Zhang et al., 2021a, 2021b).

Second, we followed the principle of evaluation system construction and extracted variables that may affect the quality of the urban blue-green landscape (Wu et al., 2020a, 2020b). These variables are derived from three aspects: socioeconomic indicators (SEI), ecological environment indicators (EEI), and landscape pattern characteristic indicators (LPCI). Next, the constituent elements of the urban blue-green landscape in Fucheng District were analyzed based on the connotations of urban development, index construction criteria, and ideas. The landscape quality evaluation index system is established with landscape quality evaluation as the target layer “*W*” and socio-economic level indicators (Pu & Cai, 2022), ecological environment indicators, and landscape pattern characteristics indicators as the standard “${U}_{i}$”. as the first-level indicator. Additionally, 29 influencing factors were extracted to form the index layer “${V}_{ij}$,” which constituted the second-level indicators of the evaluation index system (See Table 2 for details). Through hierarchical analysis (AHP), the initial weights of the indicators were calculated, and the blue-green landscape quality evaluation index system of Fucheng District was determined (He et al., 2020; Liu et al., 2021). These variables and weights constitute our indicator system for evaluating landscape quality, which can comprehensively reflect some influencing factors such as the level of urban development, ecological conditions, and landscape pattern characteristics.

**Table 2:** Landscape quality assessment indicator systems (self-drawing)

Target layer *W*	Standard layer ${{\varvec{U}}}_{{\varvec{i}}}$	Indicator layer ${{\varvec{V}}}_{{\varvec{i}}{\varvec{j}}}$	Weighting
Landscape Quality Composite Index (LQCI)	Socio-economic Index (SEI)	1. Urbanization (U)	0.907%
		2. Population scale (PS)	2.151%
		3. Population growth ratio (PGR)	0.601%
		4. Gross domestic product (GDP)	2.222%
		5. GDP growth ratio (GDPGR)	0.622%
		6. Building density (BD)	0.693%
		7. Road network density (RND)	1.369%
		8. Park number (PN)	1.102%
	Ecological Environment Index (EEI)	9. Sunshine duration (SD)	2.471%
		10. Arable area (AA)	1.547%
		11. Water area (WA)	2.293%
		12. Total water resources (TWR)	1.511%
		13. Water resources per capita (WRPC)	2.187%
		14. Forest cover ratio (FCR)	1.511%
		15. Greening rate in built-up area (GRBA)	0.764%
		16. Green space per capita (GSPC)	1.689%
		17. City park number (CPN)	2.634%
	Landscape Pattern Character Index (LPCI)	Vegetation:	
		18. Number of plaques (NP)	4.494%
		19. Density of plaques (DP)	2.841%
		20. Average plaque size (AREA MN)	1.188%
		21. Largest plaque index (LPI)	0.981%
		22. Landscape shape index (LSI)	3.771%
		23. Plaque aggregation (AI)	3.461%
		Water area:	
		24. Number of plaques (NP)	3.564%
		25. Density of plaques (DP)	0.465%
		26. Average plaque size (AREA MN)	2.324%
		27. Largest Plaque Index (LPI)	0.671%
		28. Landscape Shape Index (LSI)	2.738%
		29. Plaque aggregation (AI)	3.151%

Evaluating the quality of urban blue-green landscapes is a complex, multidimensional issue (Mu et al., 2020) that involves factors from multiple areas, such as socioeconomics, ecology, and landscape patterns. These factors interact and influence each other and cannot be measured by simply using a single indicator or method. Therefore, establishing a scientific and reasonable indicator system is a necessary condition and foundation for evaluating the quality of urban blue-green landscapes. It can comprehensively reflect the structure, function, benefits, and trends of urban blue-green landscapes, thereby providing an objective and comprehensive basis for urban landscape planning and design (Li & Lange, 2023; Wu et al., 2020a, 2020b).

After establishing the urban blue-green landscape quality evaluation index system, it was necessary to collect and process data for each index to facilitate model training and output evaluation results. To ensure the objectivity and accuracy of the data, we utilized a variety of sources, including the statistical yearbook released by the National Bureau of Statistics, environmental monitoring reports published by the Environmental Protection Department, and satellite remote-sensing data. We selected data from 2015 to 2022 as parameters for model training, encompassing changes in socioeconomic development, ecological and environmental conditions, and landscape pattern characteristics (Table 3).

**Table 3:** Input parameters for SEI, EEI model training (self-drawing)

Year	U	GDPGR	PS	PGR	CA	SD	VCR	FCR	FA	GRBA	GSPC	WA	TWR	AA	BD	RND	CPN
2015	76.00	8.50	87.56	2.30	422.00	1221.60	22	17.00	1.45	50.00	1.06	598	2.36	0.14	24.00	1.91	8
2016	76.85	8.40	90.69	4.38	597.70	1274.20	35	26.00	1.43	49.20	2.30	598	2.31	0.15	25.00	1.94	8 <
2017	77.71	9.30	92.87	4.00	597.00	1263.50	35	28.60	1.44	48.00	2.25	598	2.29	0.17	25.00	1.98	8
2018	78.75	5.70	94.72	6.50	554.00	1287.60	36	28.30	1.45	40.00	2.10	598	2.68	0.18	24.00	1.98	10
2019	81.47	8.90	96.10	4.00	597.00	1298.10	35	28.00	1.45	37.51	2.19	598	2.79	0.18	25.00	2.13	10
2020	80.25	4.90	129.85	4.13	597.30	1257.80	36	28.34	1.45	38.27	1.61	598	2.84	1.68	24.00	2.40	10
2021	74.30	9.00	131.03	4.13	554.47	1269.80	36	28.30	1.23	37.00	1.48	598	2.21	1.68	22.57	2.34	12
2022	82.00	5.00	131.03	3.15	564.12	1289.70	35	36.10	1.52	37.89	1.53	598	3.53	1.68	23.00	2.45	12

List of abbreviations:*U* urbanization **(%)**, *GDPGR* GDP growth rate **(%)**, *PS* population scale (km^2^)**,** *PGR* population growth rate (‰), *CA* city area (km^2^), *SD* sunshine duration (h), *VCR* vegetation coverage rate (%), *FCR* forest coverage rate (%), *FA* forest area (million hm^2^), *GRBA* greening rate in built-up areas (%), *GSPC* green space per capita (m^2^), *WA* water area (km^2^), *GWR* gross water resource (billion m^3^), *AA* arable area (million hectares), *BD* building density (%), *RND* road network density (km/km^2^), *CPN* city park number

- 4. Assessment criteria and level

For landscape quality assessment, reasonable assessment criteria and rating are crucial in the equation for calculating the composite index value for the quality of the landscape, as follows:

1 $${\text{LQCI}}=\sum_{j=1}^{n}{w}_{j }\times {V}_{ij}$$

where LQCI represents the Landscape Quality Composite Index, ${w}_{j}$ is the indicator *j* criterion weight, ${V}_{ij}$ is the degree of normalization of the *j*th criterion in year *i*, and *n* is the number of indicators (Bera et al., 2022; Zhang et al., 2021a, 2021b).

The calculation process of this equation involves the weighting and normalization of different indicators, thus integrating the contribution of each indicator to landscape quality. By weighting and summing the different indicators, we can obtain a composite landscape quality index that helps assess landscape quality changes in different years in a more comprehensive way (Hu et al., 2021). This approach can better reflect the impact of different indicators over time, thereby providing a more accurate assessment of landscape quality.

The Landscape Quality Composite Index (LQCI) was categorized into four levels (See Table 4 for details) based on references to relevant studies and the current urban landscape pattern and ecological environment of Fucheng District: excellent quality at the level I, good quality at level II, fair quality at level III, and acceptable at level IV (Priya & Senthil, 2021; Bera et al., 2022).

**Table 4:** Blue-green landscape quality level assessment criteria (self-drawing)

QL	Score	Blue and green landscape features
I	10–8	Complete structure and rich types; strong ecological service functions; a high percentage of good air days; high forest coverage; high green space rate; high total water catchment and water resources, high level of water resources per capita
II	8–6	Structural integrity with strong ecological services; a high percentage of good air days; high forest cover; good level of green space; abundant water resources, with a high per capita occupancy rate
III	6–4	The structure is relatively complete and meets the basic ecological service functions; the forest coverage and green space rate levels are up to standard; water resources meet the production and living of the city
IV	4–2	Structural functions to be improved to meet the basic ecological service functions; forest coverage, green space rate, and water resources level basically meet the standards

The level calculation equation is as follows:

2 $$Level=ceil\left(\left(x-{S}_{1}\right)/\left({S}_{n}-{S}_{1}\right)\times n\right)$$

where *x* is the combined urban landscape quality value; ${S}_{1}$ and ${S}_{n}$ are the lowest and highest scores, respectively, and *n* is the number of levels of the required score; ceil denotes the upward rounding function that rounds the calculated result to the nearest integer.

- 4. GA-BP neural network modeling

First, we establish the GA-BP neural network path based on the basic architecture of the BP neural network, which consists of an input layer, a hidden layer, and an output layer.

**Fig. 4:**
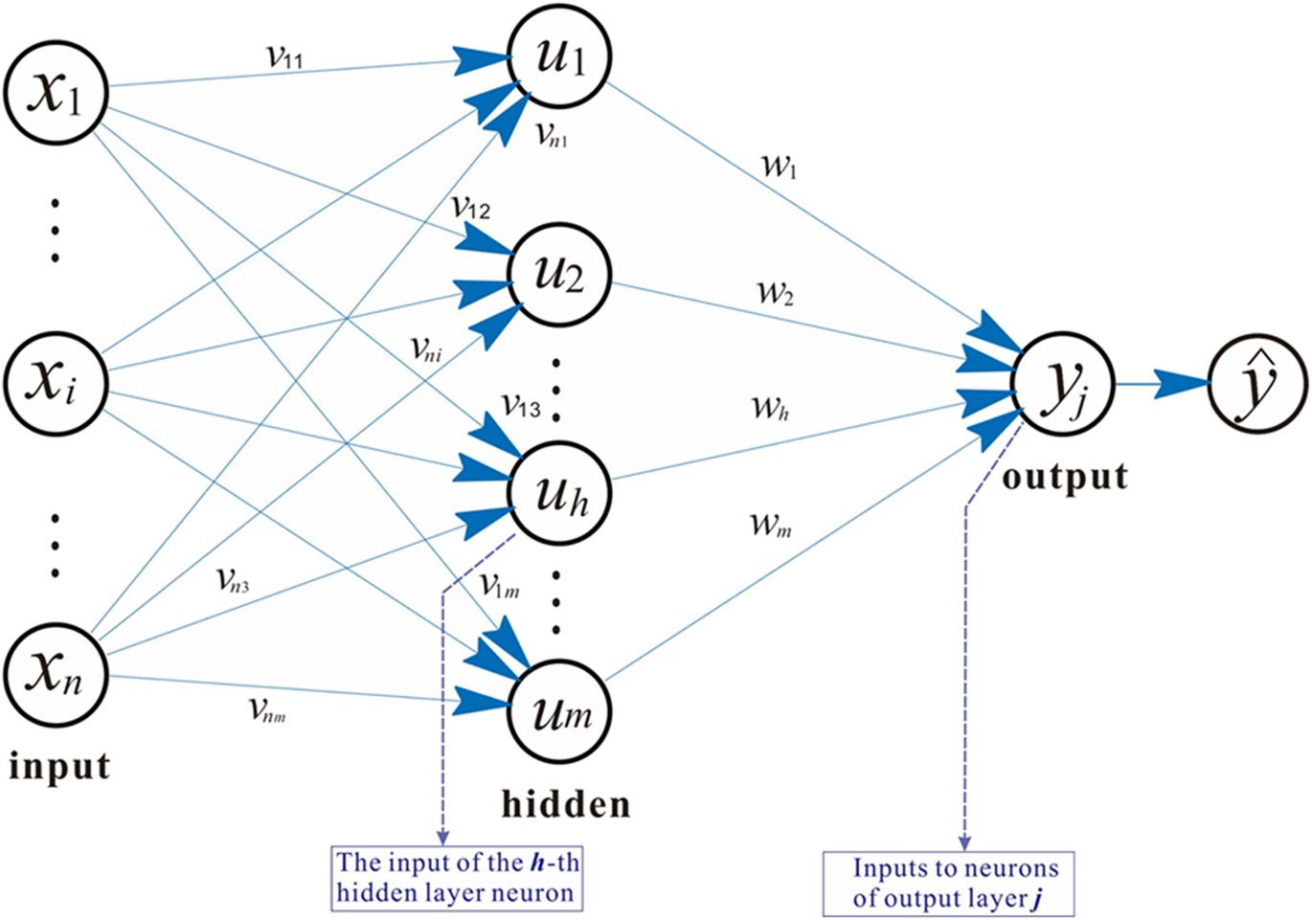
BP neural network structure (self-drawing)

We used a BP neural network to model the urban landscape quality assessment problem. The BP neural network consists of an input layer (${x}_{1\dots \dots }{x}_{n}$), hidden layer (${u}_{1}\dots \dots {u}_{m}$), and output layer (*y*), as shown in Fig. 4. The input layer receives the data we want the network to learn from, such as socioeconomic and ecological indicators. The output layer produces the result that the network learns, such as the landscape quality composite index. The hidden layer helps the network process the data and find patterns.

Each neuron in the network has a weight, bias, and activation function. The weight is a number that indicates how much influence one neuron has on another neuron. The bias is a number that indicates how easily a neuron can be activated. The activation function indicates how the neuron should calculate its output from its input (Zhang et al., 2021a, 2021b). We used a sigmoid function as the activation function, which takes continuous values within (0,1) and ${\varvec{f}}\left(x\right)$**=**$\frac{1}{1+{{\varvec{e}}}^{-{\varvec{x}}}}$ (Liu et al., 2010) where *e* is the base of the natural logarithm, and *x* is the weighted input to the neuron (the weighted input is equal to the input multiplied by the weights and added to the bias).

To train the network, we used a set of training samples ${x}_{n}$ = ($n=1, 2,\dots z$) where *z* is the number of samples and ${x}_{n}$= (${x}_{n1},{x}_{n2},\dots {x}_{nm}$), v is the input weight vector of m input value for each sample. A vector of the expected output values ${y}_{j}$ exists for each sample. We initialized the weights and biases randomly and set the training error allowance *R*. We then calculated the network output by multiplying the value of each layer by the corresponding weight and adding the bias variable. An activation function was applied to obtain the final output. 

We compared the network output with the expected output and calculated the error indicator function ${\text{E}}=\frac{1}{2}\sum_{n=1}^{n}{\Vert {y}_{j}-\widehat{y}\Vert }^{2}$ where $\widehat{y}$ is the vector of actual network output. The error is then reduced by slightly changing the weights and bias using a learning algorithm called backpropagation. This process is then repeated several times with different data until the error is less than *R* or the maximum number of iterations is reached (Wu et al., 2020a, 2020b). Using this method, we aimed to obtain a neural network that can accurately evaluate urban landscape quality based on various indicators.

**Fig. 5:**
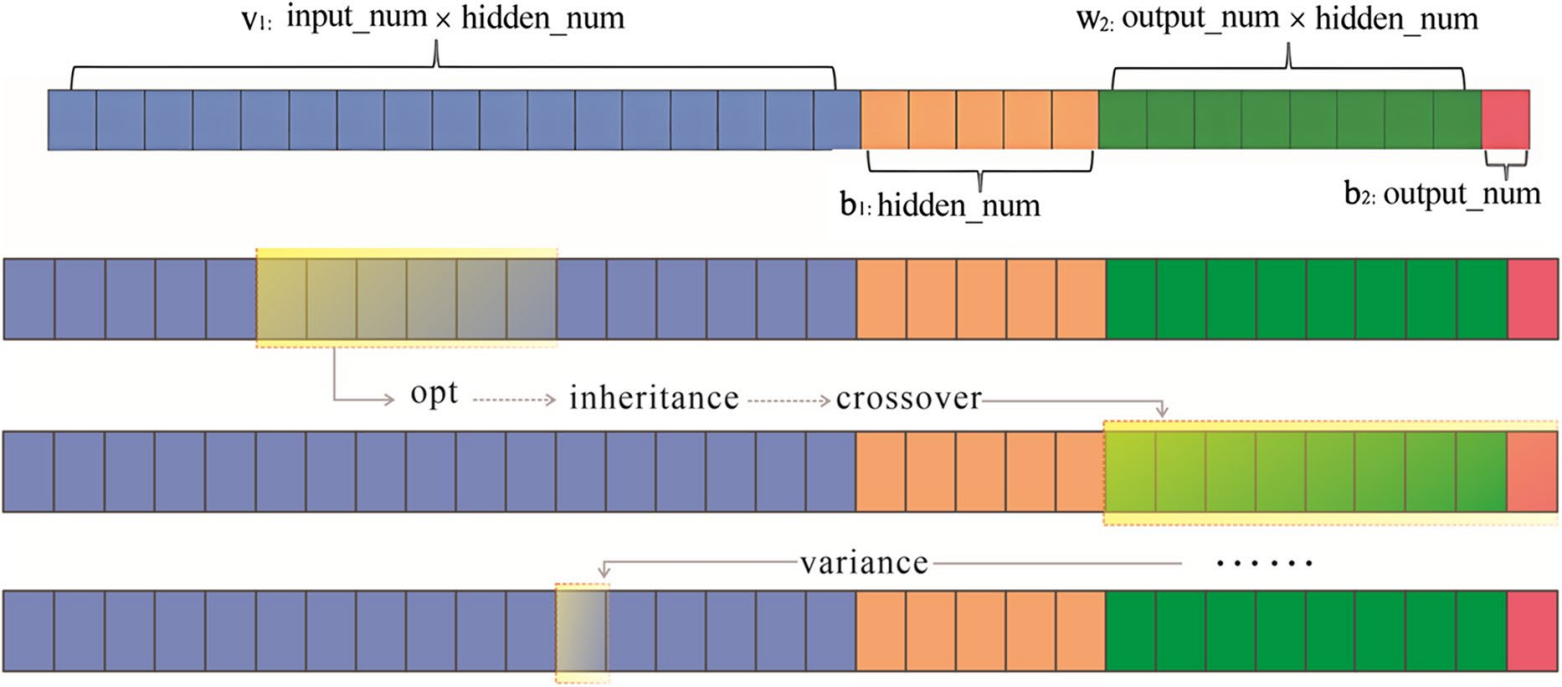
Population crossover and variation processes (self-illustration)

Next, we used a genetic algorithm to optimize the initial weights and thresholds of the network and trained the network with the backpropagation algorithm until it converged to the desired accuracy (Ye et al., 2018; Xu et al., 2022). The genetic algorithm that we used treated all parameters as flat chromosomes, as shown in Fig. 5, to optimize the initial weights of the BP network.

Genetics has been applied to neural networks to guide weight optimization and topology selection. In this study, genetic algorithms were used to determine the initial weights of the network. The connection weight optimization problem for the preferred neural network is described as follows:

3 $${\text{Min}}\left({\text{E}}\right)={\text{f}}\left({{\text{w}}}_{1},{{\text{w}}}_{2}\dots \dots {{\text{w}}}_{{\text{n}}}\right)$$

Equations ${{\text{w}}}_{1}$,${{\text{w}}}_{2}$,……${{\text{w}}}_{{\text{n}}}$ are the connection weights after uniform numbering (including the connection weights of the input nodes, intermediate layer nodes, intermediate layer nodes, and output layer nodes); *n* is the total number of connection weights, and the constraint is − 1 < ${{\text{w}}}_{{\text{i}}}$  < 1. The genetic process is as follows:

- 1. Set up to generate an initial population where a chromosome corresponds to a set of network weights and calculate the fitness function for each population 1. Set the number of chromosome populations *N*, number of generations gen, crossover probability ${{\text{P}}}_{{\text{e}}}$, and mutation probability ${{\text{P}}}_{{\text{m}}}$ and calculate the fitness value for each chromosome. Let the input data be *x* and the true output be *y*. After the model predicts the output as $\widehat{{\text{y}}}$, ${{\text{v}}}_{1}$ (the matrix transformation of the front part of chromosome ${{\text{v}}}_{1}$) is the weight lift of the connected input and hidden layers, ${{\text{b}}}_{1}$ is the deviation of the connected input and hidden layers, ${{\text{w}}}_{2}$ is the weight matrix of the connected output and hidden layers, and ${{\text{b}}}_{2}$ is the deviation of the connected output and hidden layers.
- 2. Evaluate the fitness function of the previous population to determine the probability of selection, with a higher fitness indicating a greater likelihood of being chosen. Through crossover and mutation operations, genetic diversity increases within the population, and individuals with better fitness may emerge in subsequent iterations.
- 3. Using a roulette wheel selection method, chromosomes are selected from the population, and crossover operations are performed with a probability of ${{\text{P}}}_{{\text{e}}}$. Additionally, mutated genes can be added to crossed chromosomes with a mutation probability of ${{\text{P}}}_{{\text{m}}}$, with the weights of mutated genes randomly adjusted by a value ≥ 1(Lei & Jain, 2022).
- 4. After the genetic variation process, new chromosomes constitute the next-generation population, and the iteration process continues until the chromosome with the highest fitness value is identified as the optimal solution.
- 5. Using a genetic algorithm, determine the optimal chromosome decomposition, connection weights, and thresholds, which can be used as initial values for the BP neural network model. The network is trained until the convergence accuracy criteria are met using the BP algorithm for local optimization based on the GA global search for optimal solutions (Ye et al., 2018; Jiao et al., 2023; Nafi’Shehab et al., 2021).

Subsequently, to better evaluate the performance of our model, we designed a network structure that divided the dataset into training, validation, and test sets. The training set was used to train the model, the validation set was used to adjust the model parameters, and the test set was used to evaluate the generalization ability of the model.

**Fig. 6:**
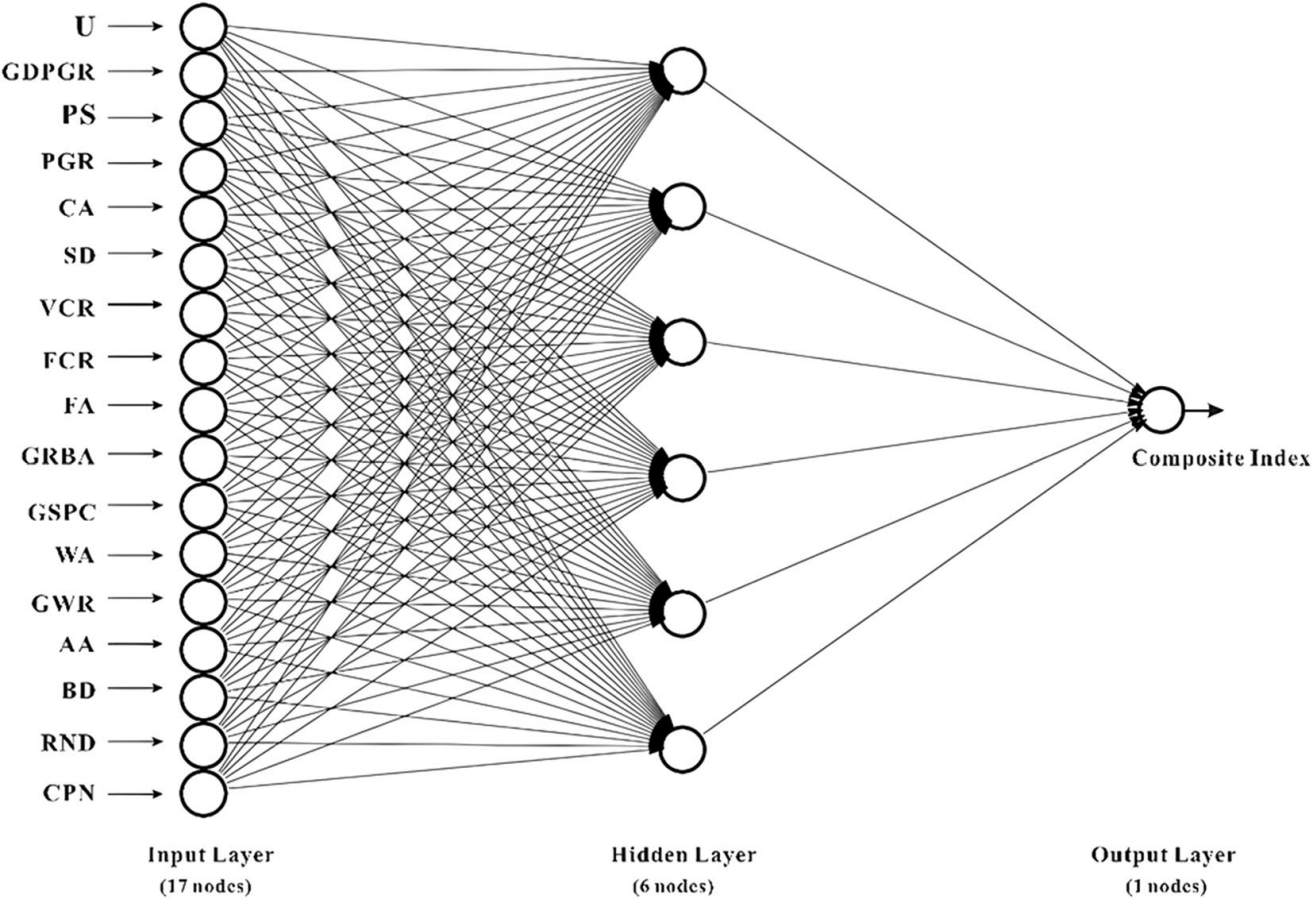
Neural network structure for landscape quality assessment (self-drawing)

After identifying the samples, we designed a neural network model. Urban landscape quality assessment can be considered a pattern recognition problem in which the neural network aims to accurately evaluate the composite index based on the input vectors. The topology of a neural network model is a crucial factor that determines the input, output, and layers of the network. The network is repeatedly trained using different learning rates and numbers of hidden layers to minimize the number of iterations and systematic errors of the network until it becomes stable (Wang et al., 2020a, 2020b). Finally, the network parameters, structure, and evaluation model structure were determined, which consisted of a three-layer BP neural network with 17 input nodes, six hidden nodes, and one output node (as shown in Fig. 6). Input indicators are variables that can reflect the level of the ecological environment, such as population size, urbanization rate, green space rate, and total water resources. The hidden layer neurons are variables that can reflect the level of urban development and ecological environment and can reflect the spatial structure, morphology, and function of the landscape, such as the number of patches, the density of patches, and the average patch area. The output indicator is the landscape quality composite index, which comprehensively evaluates the quality of urban blue and green landscapes, the larger the index, the higher the quality.

We used hierarchical analysis to determine the weights of each indicator and normalized the data such that its value domain was unified as [0, 1] to be used for model training (Sun et al., 2017a, 2017b). Then, a genetic algorithm was used to optimize the BP neural network model, the output indicators were calculated based on the input indicators and hidden layer neurons, and the error was compared with the real data to obtain the optimal model parameters and prediction results (Nafi’Shehab et al., 2021).

- 6. Model validation

Because of the small sample size, we used the leave-one-out method to validate the reliability of the model. Leave-out cross-validation (LOOCV) is a special cross-validation method that trains on a sample of data and repeats it *N* times, obtaining one prediction error each time. The *N* prediction errors are averaged to obtain the final prediction error. The prediction error is the difference between the model’s predicted composite value and the actual composite value, which helps us to understand the model’s performance on unknown data and thus assess the model’s generalization ability (Wild et al., 2022). The equation used is as follows:

4 $${{\text{CV}}}_{({\text{n}})}=\frac{1}{{\text{n}}}\sum_{{\text{i}}=1}^{{\text{n}}}{{\text{MSE}}}_{{\text{i}}}$$

where *n* is the number of samples in the dataset, and ${{\text{MSE}}}_{{\text{i}}}$ is the mean square error at the *i*th time when the *i*th sample is left as the test set. In general, the smaller the prediction error, the better the model. This method can help improve the reliability and validity of research results and provide more references and lessons for subsequent research (Zeng et al., 2022).

## Results and analysis

### Characterization of blue-green landscapes

Based on satellite images and Fragstats software analysis and calculation, that is, number of patches (NP), patch density (PD), average patch area (AREA_MN), maximum patch index (LPI), landscape shape index (LSI), and patch agglomeration index (AI), which can characterize the distribution, morphology, and size features of the blue-green landscape patches, which provide an important basis for assessing the quality of the city’s blue-green landscape.

As shown in Table 5, we can observe that the green and water landscape features in urban areas have undergone significant changes. The green area (NP) has experienced significant growth during this period, expanding from 175 in 2015 to 29,565 in 2022. This indicates a significant expansion trend in green spaces in urban areas, which may reflect improvements in urban planning policies aimed at creating more diverse green spaces. Further analysis showed that the Landscape Shape Index (LSI) also showed an upward trend, indicating that the shape of greenery is becoming more diverse and complex. At the same time, the Average Green Area (AREA MN) and Landscape Aggregation Index (AI) also showed fluctuating growth, indicating that greenery is distributed more widely and compactly. This helps provide larger green units to meet residents’ leisure needs or ecosystem services.

**Table 5:** Green and Water Landscape Index (GWLI), 2015–2022 (self-drawing)

Green land							Water area					
Year	NP	PD	LPI	LSI	AREA MN	AI	NP	PD	LPI	LSI	AREA MN	AI
2015	175	0.3146	0.1854	29.6322	26.9731	172.0555	46	0.0827	2.1345	12.8114	42.9163	91.9491
2016	677	0.020	0.0552	34.74679	1.10415	18.14000	748	0.0100	0.3847	21.3263	2.08021	37.4000
2017	708	0.020	0.0527	35.24325	0.99122	70.45963	720	0.0100	0.3842	21.0360	2.12301	24.4731
2018	664	0.020	0.0471	33.62920	1.00046	63.86000	696	0.0100	0.3852	20.7032	2.17359	27.4731
2019	567	0.020	0.0423	33.4561	1.00120	64.95010	656	0.0100	0.3719	20.8791	2.16781	27.4512
2020	172	0.3092	3.0134	20.0533	68.2914	175.7685	34	0.0611	2.9726	12.5533	59.5085	92.2395
2021	26,432	21.87	0.3210	254.321	0.82820	181.4021	215	0.1623	0.3123	18.7456	6.04320	95.2010
2022	29,565	21.991	0.4006	273.5354	0.84450	181.4137	229	0.1703	0.3224	19.1082	6.41520	95.2605

Similarly, the water landscape has undergone significant change. The water area (NP) has increased from 46 in 2015 to 229 in 2022, indicating that the scale of urban water bodies has also undergone significant expansion. Similar to greenery, the Landscape Shape Index (LSI), Average Water Area (AREA MN), and Aggregation Index (AI) of water bodies also showed a growth trend. This means that there are more water units with diverse shapes and increasingly concentrated distributions. This trend may be related to the implementation of urban ecosystem protection and landscape beautification policies to improve the attractiveness and sustainability of urban water landscapes.

In summary, from 2015 to 2022, there have been significant changes in the distribution, shape, and scale of the Greenland and water bodies. The number of green areas has increased rapidly, and their scale is continuously expanding. This may be closely related to urban environmental governance and development. However, the number of water bodies fluctuated with relatively stable shapes and slightly expanded scales. According to statistical data, comparing 2015 and 2022, the construction land area of Fucheng District increased significantly, and the proportion of the total area increased from 40.2 to 52.1%, reflecting the rapid urbanization of Fucheng District. At the same time, the area of arable land increased from 0.14 to 16.8 million ha, and the areas of forest land, grassland, water area, wetland, etc., also increased substantially, reflecting the changes in the land use structure of Fucheng District (National Platform for Integrated Earth Observation Data Sharing). These changes may be influenced by various factors, such as urban planning, environmental policies, social needs, natural disasters, and human interference (Cong et al., 2019). These changes may affect the quality of urban blue-green landscapes and ecosystem services but require further scientific evaluation and prediction (Bian et al., 2023).

### Measurement and output of urban blue-green landscape quality

By analyzing the characteristics of urban blue-green landscapes, we can understand their changing trends and features. However, these characteristics do not directly reflect the quality level and evaluation results for urban blue-green landscapes. To assess the quality of urban blue-green landscapes more comprehensively and objectively, we need to combine the characteristics of urban blue-green landscapes with social and economic factors and consider multiple dimensions, such as the function, benefit, and value of urban blue-green landscapes (Wang & Zhang, 2023), including social, economic, and landscape information from Fucheng District from 2015 to 2022. Therefore, this section combines the evaluation index system, assessment criteria, neural network structure model, and deep learning algorithm to build, train, and test the neural network using the Neural Network Genetic Algorithm Toolbox of MATLABR2020b software and outputs and assesses the quality level of urban blue-green landscapes.

First, we randomly divided the dataset into three parts: 70% of the data was used as the training set, 15% as the validation set, and the remaining 15% as the test set. After repeated training, the fitness value of BP training was obtained. Subsequently, the network function @myNeuralNetworkFunction for the MATLAB Compiler was exported and saved. Finally, the function and rand (1, 29) were used to predict the results of these 29 data, and a genetic algorithm was initiated.

We then set the genetic algorithm fitness function @myNeuralNetworkFunction and the number of variables, Ib lower limit *x* ≥ 0 and ub upper limit *x* ≤ 10, the number of populations *N* is 20, the number of genes is 100, the crossover probability ${{\text{P}}}_{{\text{e}}}$ and mutation probability ${{\text{P}}}_{{\text{m}}}$ are retaken as 0.3 and 0.1, respectively, and the numerical crossover, mutation, and iterative process of finding the best.

**Fig. 7:**
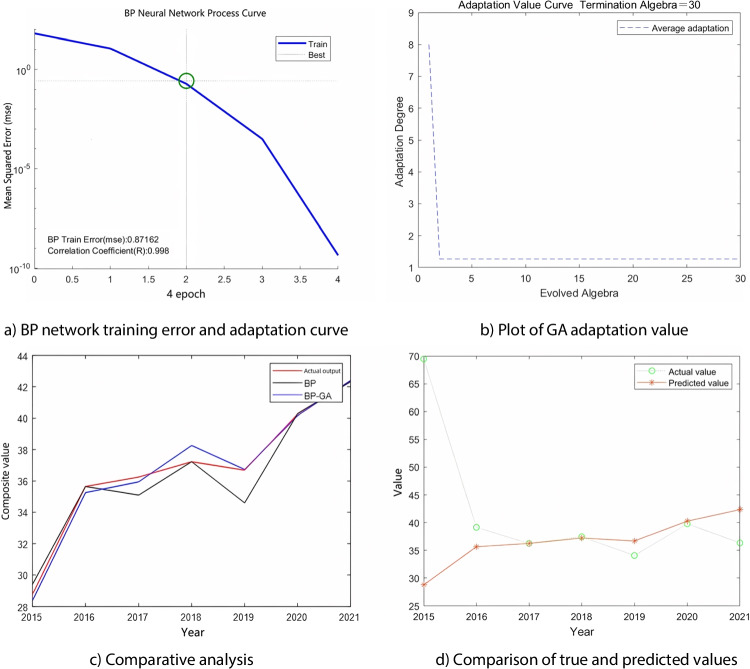
The process of data prediction and optimization (the above picture is self-drawn)

The BP network was trained with a precision error of 0.001 and a learning rate of 0.001. After repeated training, the BP training error (MSE) was 0.8716, and the correlation coefficient (*R*) was 0.998, indicating a good fitting effect (Fig. 7a). The mean absolute error (MAE) was 0.56017, and the mean relative percentage error (MAPE) was 1.5988%. For comparison, the same parameters were used to train and test the standard BP neural network. During GA selection, crossover, and variation, individuals are continuously updated to determine the optimal solution. As the number of iterations increases, the maximum fitness value in the population tends to stabilize or increase, and the mean squared difference in fitness values in the population tends to decrease or stabilize (Bera et al., 2022; Pu & Cai, 2022). The fitness of the chromosomes tended to stabilize after 30 iterations of search by the GA (as shown in Fig. 7b). The root mean squared error (RMSE) after the BP-GA training was 0.46135, mean squared error (MSE) was 0.21285, mean absolute error (MAE) was 0.33483, and mean relative percentage error (MAPE) was 0.94772%. Figure 7c, d, the actual, BP-predicted, and BP-GA-optimized values of the combined urban landscape quality values.

On the one hand, after calculating the LQCI according to Eq. 1, the BP and GA-BP models were used for prediction. The results showed that both models could fit and predict the comprehensive urban blue-green landscape quality index. From a comparison of the actual and predicted values, it can be seen that both models can capture the changing trend and fluctuation characteristics of the urban blue-green landscape quality comprehensive index well. However, the predicted value of the GA-BP model is closer to the actual value, with smaller errors, higher correlation, better performance, and higher accuracy and stability (Table 6). This indicates that the use of genetic algorithms to optimize the initial weights and thresholds of the BP neural network model can improve the model’s fitting degree and sensitivity. At the same time, optimizing the parameters of the BP neural network model can improve its performance and adaptability, thus avoiding problems such as local optima or overfitting, and making the model more accurately reflect the actual situation of urban blue-green landscape quality (Ye et al., 2018; Nafi’Shehab et al., 2021).

**Table 6:** Comparison and ranking of actual values and simulation outputs for the combined urban landscape environmental quality value (self-drawing)

Year	Truth value (LQCI)	BP predicted (LQCI)	GA-BP optimization (LQCI)	Level
2015	28.77	29.4074	28.3763	IV
2016	35.65	35.6488	35.2576	III
2017	36.25	35.0981	35.9475	III
2018	37.23	37.2289	38.2652	II
2019	36.69	34.6001	36.7376	II
2020	40.27	40.2912	40.1617	I
2021	42.34	42.3212	42.4046	I
2022	43.72	36.6588	41.4849	I

However, further tests are required to verify the reliability of the model and the results. Based on the sample size (2015–2022), we used the Cvpartition function in MATLAB to partition the dataset and implemented leave-one-out cross-validation using the Crossvalind function. The performance metrics of the model for the training, validation, and test sets were evaluated by calculating the mean square error (MSE) and goodness-of-fit (*R*) values (based on Eq. 4). The results show that the model has a good fit for the training data and a low prediction error on the test set (MSE of 1.2881). In addition to MSE, *R* is also an indicator of the model performance (Zeng et al., 2022). According to the results of the goodness-of-fit values, most of the subsets have high *R* values (close to 1), indicating that the model is a good fit for the training data. Overall, the validation results can be used as a reference to assess the performance of the model; however, further analysis and judgment are needed.

Furthermore, as shown in Fig. 7 (trend of urban blue-green landscape quality comprehensive value), the *y*-axis represents the comprehensive value of urban blue-green landscape quality, and the *x*-axis represents the research period (2015–2022). According to the model, the urban blue-green landscape quality has shown a fluctuating upward trend over the years, indicating that under the strong promotion and implementation of local government policies on ecologically livable cities and the enhancement of people’s environmental awareness, the urban landscape environment quality in the study area is moving towards a higher level. Furthermore, as can be seen from Table 6 (evaluation results of urban blue-green landscape quality level), the GA-BP neural network model comprehensively calculates and predicts the urban blue-green landscape quality level from 2015 to 2022 and captures its trends and fluctuation characteristics. Compared with the traditional BP neural network model, the GA-BP neural network model has a higher prediction accuracy and lower error values (Li et al., 2019), indicating that the GA-BP neural network model is an effective evaluation method that can accurately reflect changes in urban blue-green landscape quality.

### Factors affecting urban blue-green landscape quality

The GA-BP neural network model is an effective instrument for evaluating and predicting urban blue-green landscape quality. This tool enabled us to discern the trend of landscape quality within our study area, which has notably improved from 2020 to 2022, surpassing the preceding 5 years. This outcome mirrors the comprehensive quality of urban blue-green landscapes. As indicated by prior measurements, the quality of these landscapes is influenced not only by their inherent characteristics such as distribution, shape, and scale but also by a broad spectrum of social, environmental, and economic factors (Wang et al., 2023). A holistic consideration of these factors will enhance our understanding of the mechanisms affecting urban blue-green landscape quality and provide a crucial foundation for their assessment.

From a social standpoint, factors such as the urbanization rate, population size, infrastructure level, and policymaking significantly influence the quality of urban blue-green landscapes (O’Donnell et al., 2021). Urban expansion driven by urbanization and population growth exerts pressure on natural resources and the environment. While infrastructure improvements augment city functionality and convenience, they may also disrupt city aesthetics and the ecological balance. The rationality and effectiveness of policymaking can have either positive or negative impacts on the urban blue-green landscape quality.

Economically, the GDP growth rate, income level, and consumption structure are key determinants of urban blue-green landscape quality. While the GDP growth rate signifies the city’s development level and economic vitality, it may also lead to resource consumption and environmental pollution. Income level and consumption structure reflect residents’ living standards and demand structures, influencing their perception of urban blue-green landscape quality, as well as their contribution to its protection and improvement.

From an environmental perspective, factors such as vegetation coverage, water resources, and climate change significantly impact the urban blue-green landscape quality (Zhang et al., 2022). Vegetation coverage is a crucial indicator of the city’s greening level and ecological service function. Water resources form the core element of the urban blue landscape. Climate change is a global environmental issue that affects precipitation patterns, temperature fluctuations, and disaster occurrences in cities, thereby influencing the urban blue-green landscape quality.

In summary, factors influencing urban blue-green landscape quality are multifaceted and complex. They necessitate a comprehensive consideration of various dimensions, along with their interactions and impacts. Each component of the structure of urban blue-green landscape quality represents a factor that influences the comprehensive evaluation of urban landscape quality (Wu et al., 2021). By analyzing these factors, we can identify the key determinants affecting urban blue-green landscape quality evaluation and provide scientific guidance for future urban landscape planning and design.

## Discussion

The urban blue-green landscape quality plays a vital role in the sustainable development of modern cities. Therefore, it is important to thoroughly assess and understand the key factors in this field (Song et al., 2022; Wu et al., 2021). This study adopted the genetic algorithm backpropagation neural network method to evaluate urban blue-green landscape quality in the Fucheng area of China. We established an evaluation index system that includes 29 multidimensional influencing factors that could comprehensively capture the diverse characteristics of urban blue-green landscapes. Through optimization of the genetic algorithm, we successfully reduced the training and prediction errors and improved the accuracy and efficiency of the comprehensive value evaluation of landscape quality.

In this study, we chose the genetic backpropagation neural network method because of its excellent performance in large-scale data processing and pattern recognition (Li et al., 2019). This method provides a novel way for research to comprehensively understand the various dimensions of urban blue-green landscape quality. In terms of the result analysis, we explored the trends and changes in the results between different years. The increase in greenspace area, forest coverage, and per capita water resources promoted improving urban blue-green landscape quality from 2015 to 2022. These trends were positive, but we also realized that urban blue-green landscapes faced some challenges, such as a high population growth rate, low greening rate of built-up areas, and insufficient water bodies and green space patch aggregation index. These issues must be addressed in future urban planning to continue to improve the quality and ecological service function of urban blue-green landscapes (Bian et al., 2023). We used the leave-one-out cross-validation method to verify the reliability of the model, and the results showed that the model had a low mean square error (MSE) and high goodness of fit, which proved its excellent performance. This method provides a scientific basis for urban planning and design and provides more comprehensive information for decision-makers (Wu et al., 2022).

Additionally, this study also provided a comprehensive perspective, which helped to better understand and quantify the quality of urban blue-green landscapes and offered strong support for urban planning and design. The results showed that urban blue-green landscape quality was affected by multiple factors, and land use, population density, and green space coverage played key roles in this process, indicating the importance of considering ecological, social, and economic factors in landscape quality assessment (Wang & Zhang, 2023), which also highlighted the non-linear and complex characteristics of urban blue-green landscape systems.

However, this study has some limitations. First, the data used in the analysis were limited to public sources, which might not fully capture all relevant factors affecting urban blue-green landscape quality. Future research could consider more comprehensive data sources, such as specific information from local governments or urban planning departments (Jie, 2022). Second, our study did not consider the subjective perception of residents and visitors (Hong et al., 2022), which could provide insights into the experience and preference of behavioral agents related to urban blue-green landscapes (Han & Leng, 2022; Pan et al., 2020). Finally, this study focused on a specific geographic area and period, limiting the generalizability of the results. Future research can consider expanding the research scope, including different regions and periods, to more comprehensively understand the changing trends and influencing factors of urban blue-green landscape quality (Liu et al., 2023; Mu et al., 2020).

Although this study adopted rigorous data processing and statistical analysis methods to improve the reliability of the research results, it should be noted that the use of a GA-BP neural network for landscape quality assessment is a relatively new method, and its effectiveness in different contexts remains to be further investigated. Future research could compare the results of this method with traditional methods to evaluate its reliability and validity (Jiao et al., 2023; Xu et al., 2022).

Furthermore, it is worth emphasizing that this study adopted a multidimensional landscape quality assessment method, consistent with previous research results, highlighting the importance of considering ecological, social, and economic factors. The GA-BP neural network model had higher accuracy and comprehensiveness in assessing urban blue-green landscape quality than the traditional methods. It combines the advantages of pattern recognition and large-scale data processing, thereby improving the accuracy and efficiency of landscape quality assessments (Yang, 2020).

In future research, cross-city comparison can be considered by selecting multiple cities as research areas to expand the geographic scope, better understand the differences in influencing factors, and enhance the universality of research results (Xu, 2023). In addition, exploring other optimization algorithms, such as particle swarm optimization and ant colony optimization, can further improve the performance of the neural network model and the accuracy of landscape quality assessment (Li et al., 2022; Peng et al., 2019). Using survey or interview methods will help to judge landscape quality more comprehensively and provide strong support for quantitative analysis. These research directions will continue to deepen the field of landscape quality assessment and provide more support and guidance for the sustainable development of cities.

## Conclusion

In summary, this study verifies the effectiveness and rationality of a neural network model for urban landscape quality assessment. The GA-BP neural network method exhibited higher accuracy and efficiency in large-scale data processing and pattern recognition. These research results help to deeply understand the factors affecting landscape quality and provide scientific guidance for urban planning and design. However, this study still has some limitations, including the data sources, subjective perception, and generalizability of the research results. Future research should strive to overcome these limitations and further explore the potential of alternative optimization algorithms and cross-city comparative analysis. We believe that this study has important reference value for future urban planning and design work and helps enhance the ecological benefits of urban blue-green landscapes, thereby promoting the sustainable development of cities. We look forward to future research that will continue to explore new methods and ideas to more comprehensively and deeply assess and plan the quality of urban blue-green landscapes to meet the growing urban development needs.

## Data Availability

The original dataset collected for this study can be accessed on platforms such as the Sichuan Provincial Statistical Database, China's Third National Land Survey Database, and the Juhui Database.

## References

[CR1] Bera D, Kumar P (2022). Assessing the impact of urbanization on surface runoff using vegetation-impervious surface-soil (V-I-S) fraction and NRCS curve number (CN) model. Modeling Earth Systems and Environment.

[CR2] Bian J, Chen W, Zeng J (2023). Ecosystem services, landscape pattern, and landscape ecological risk zoning in China. Environmental Science and Pollution Research.

[CR3] Cecchini M, Zambon I, Pontrandolfi A, Turco R, Colantoni A, Mavrakis A, Salvati L (2019). Urban sprawl and the ‘olive’ landscape: Sustainable land management for ‘crisis’ cities. GeoJournal.

[CR4] Cheng, Y.T., & Wu, C.G. (2020). Research progress of urban blue-green space planning approach based on local climate optimization. *Chinese Journal of Applied Ecology, 31*(11), 3935–3945. 10 13287/j 1001 9332 202011 014. 10.13287/j.1001-9332.202011.014 33300745

[CR5] Chi, Z., Guo, Y., Lai, M., (2021). A review on the development and application of artificial neural network modeling. *Computer Engineering and Applications, 57*(11), 57–69. https://kns.cnki.net/kcms/detail/11.2127.TP.20210402.1348.004.html

[CR6] Cong P, Chen K, Qu L, Han J (2019). Dynamic changes in the wetland landscape pattern of the Yellow River Delta from 1976 to 2016 based on satellite data. Chinese Geographical Science.

[CR7] Han B, Leng H (2022). Impacts of green space on the subjective well-being of elderly people in a cold-land community: A case study of Changchun City. Landscape Architecture.

[CR8] He, H., Wu, Q., Yang, S., Yang, F., Li, B., Zhu, Y., & Ma, S. (2020). Construction of a functional indicator system for soil and water conservation resilient landscapes. *Yellow River, 42*(12). 10.3969/j.issn.1000-1379.2020.12.01

[CR9] Hong XC, Huang S, Wang GY, Liu J (2022). Long-term perception modeling of soundscape in urban parks: A case study of three urban parks in Vancouver Canada. Landscape Architecture.

[CR10] Hu, W., Li, W., Wang, L., Suo, Y., Sun, X., Li, J., & Chen, Q. (2021). Health assessment of small and medium-sized rivers based on GA-BP optimization model. *Journal of Ecology, 3*, 18. https://www.fx361.com/page/2021/0407/15853994.shtml

[CR11] Hua X, Zhang H, Wu X, Zhao J (2020). Study on evaluating urban park landscape quality based on a BP neural network optimized by genetic algorithm. Ecological Indicators.

[CR12] Jahani A, Allahverdi S, Khosravi H, Khorasani N (2022). Environmental modeling of landscape aesthetic value in natural urban parks using artificial neural network technique. Modeling Earth Systems and Environment.

[CR13] Jiao, L., et al. (2023). An assessment model for urban resilience based on the pressure-state-response framework and BP-GA neural network." *Urban Climate, 49*: 101543. 10.1016/j.uclim.2023.101543

[CR14] Jie, Q. (2022). Precision and intelligent agricultural decision support system based on big data analysis. *Acta Agriculturae Scandinavica*, *Section B—Soil & Plant Science, 72*(1), 401–414. 10.1080/09064710.2021.2008477

[CR15] Ke Fu (1992). Environmental Systems Engineering.

[CR16] Leggett, J.A., & Carter, N.T. (2019). *Rio+20: The United Nations Conference on Sustainable Development*. June Retrieved from https://digital.library.unt.edu/ark:/67531/metadc93939/.

[CR17] Lei, F., & Jain N. (2022). Characteristics of plant landscape design in modern urban landscape design based on BP neural network. *In Cyber Security Intelligence and Analytics* (pp. 551–556). Springer International Publishing.

[CR18] Li L, Lange KW (2023). Assessing the relationship between urban blue-green infrastructure and stress resilience in real settings: A systematic review. Sustainability.

[CR19] Li B, Tian X, Zhang M (2019). Thermal error modeling of machine tool spindle based on the improved algorithm optimized BP neural network. The International Journal of Advanced Manufacturing Technology.

[CR20] Li Y, Yu K, Liang J, Yue C, Qiao K (2022). A landscape-aware particle swarm optimization for parameter identification of photovoltaic models. Applied Soft Computing.

[CR21] Liu Y, Wu F, Jiang H, Chen D (2010). Environmental quality assessment method based on GA-BP neural network. Computer Simulation.

[CR22] Liu Y, Zhong Y, He F-Y, Weizheng S, Qionglai Y (2021). Evaluation of landscape and environmental quality of suburban recreational forests based on the AHP method. Sichuan Forestry Science and Technology.

[CR23] Liu F, Lin B, Meng K (2023). Green space settlement landscape optimization strategy under the concept of ecological environment restoration. Journal of King Saud University-Science.

[CR24] Macsimovici, S., (2019). Blue-green solutions for urban development [PDF file]. *China construction news*, People’s Republic of China (PRC) Ministry of Housing and Urban-Rural Construction.

[CR25] Mu B, Liu C, Tian G, Xu Y, Zhang Y, Mayer AL, Kim G (2020). Conceptual planning of urban–rural green space from a multidimensional perspective: A case study of Zhengzhou China. Sustainability.

[CR26] Nafi’Shehab, Z., Jamil, N.R., Aris, A.Z., & Shafie, N.S. (2021). Spatial variation impact of landscape patterns and land use on water quality across an urbanized watershed in Bentong, Malaysia. *Ecological Indicator*s, 122, Article ID 107254.

[CR27] Nicolini E (2022). The circularity of MSW in urban landscapes: An evaluation method for a sustainable system implementation. Sustainability.

[CR28] Nikbakht S, Anitescu C, Rabczuk T (2021). Optimizing the neural network hyperparameters utilizing genetic algorithm. Journal of Zhejiang University-Science A.

[CR29] O’Donnell EC, Netusil NR, Chan FK, Dolman NJ, Gosling SN (2021). International perceptions of urban blue-green infrastructure: A comparison across four cities. Water.

[CR30] Pan Y, Weng G, Li C, Na S (2020). Measurement of location advantages of 5A scenic areas in the silk road tourism belt based on DS evidence theory. Journal of Natural Resources.

[CR31] Peng J, Zhao S, Dong J, Liu Y, Meersmans J, Li H, Wu J (2019). Applying ant colony algorithm to identify ecological security patterns in megacities. Environmental Modelling & Software.

[CR32] Priya, U.K., & Senthil, R. (2021). A review of the impact of the green landscape interventions on the urban microclimate of tropical areas. *Building and Environment, 205*, Article ID 108190. 10.1016/j.buildenv.2021.108190

[CR33] Pu, Y.P., Cai, Y.H. (2022). On the development level of green economy in different regions based on GA-BP model, *Environment and Public Health*, 1587896, 10.1155/2022/1587896 10.1155/2022/1587896 PMC9481356 36120140

[CR34] Song S, Wang S, Shi M, Hu S, Xu D (2022). Urban blue–green space landscape ecological health assessment based on the integration of pattern, process, function and sustainability. Scientific Reports.

[CR35] Statistical Yearbook Platform. (n.d.). Retrieved April 27, 2023, from https://www.yearbookchina.com/navipage-n3018090304000148.html

[CR36] Sun H, Li J, Li J (2017). Ecological evaluation of urban green space based on improved BP neural network. Journal of Intelligent & Fuzzy Systems.

[CR37] Sun H, Li J, Li J (2017). Research on the ecological evaluation of urban green space based on improved BP neural network. Advances in Intelligent Systems and Computing.

[CR38] Wang FW, Zhang YH (2023). Landscape fragmentation analysis and ecological quality assessment at watershed scale. Journal of Natural Disasters.

[CR39] Wang, S., Zhang, Y., & Liu, Y. (2020). Study on the evaluation of urban green space ecological quality based on BP neural network optimized by particle swarm algorithm. *Journal of Physics: Conference Series, 1538*(1), 10.1088/1742-6596/1538/1/012049

[CR40] Wang Y, Li X, Zhang Y, Li Y (2020). Assessing the landscape benefits of urban water bodies: A case study in Hangzhou China. Landscape and Urban Planning.

[CR41] Wang, Y., Chen, Z., & Cheng, Y. (2021). Construction of a characterization system for rural landscape features and landscape character in the new era. *Landscape Architecture, 28*(7), 107–113. 10.14085/j.fjyl.2021.07.0107.07

[CR42] Wei J, Yue W, Li M, Gao J (2022). Mapping human perception of urban landscape from street-view images: A deep-learning approach. International Journal of Applied Earth Observation and Geoinformation.

[CR43] Wild M, Behm S, Beck C, Cyrys J, Schneider A, Wolf K, Haupt H (2022). Mapping the time-varying spatial heterogeneity of temperature processes over the urban landscape of Augsburg. Germany. Urban Climate.

[CR44] Wu J, Yang S, Zhang X (2020). Interaction analysis of urban blue-green space and built-up area based on coupling model—A case study of Wuhan Central City. Water.

[CR45] Wu L, Zhou J, Li ZH (2020). Applying of GA-BP neural network in the land ecological security evaluation. IAENG International Journal of Computer Science.

[CR46] Wu L, Dong Q, Luo S, Jiang W, Hao M, Chen Q (2021). Effects of spatial elements of urban landscape forests on the restoration potential and preference of adolescents. Land.

[CR47] Wu, G., Miao, Y., & Wang, F. (2022). Intelligent design model of urban landscape space based on optimized BP neural network. *Journal of Sensors*, 2022, Article ID 9704287. 10.1155/2022/9704287

[CR48] Xu, L. (2023). Value assessment and renovation design of buildings in the perspective of urban renewal: Case studies in China and Italy. https://www.research.unipd.it/handle/11577/3474288

[CR49] Xu J, He Z, Yuan R (2016). A nonlinear contour map optimization method based on LM-BP neural network. China Mechanical Engineering.

[CR50] Xu L, Liu K, Sang K, Lin G, Luo Q, Huang C, Giordano A (2022). Assessment of the exterior quality of traditional residences: A genetic algorithm–backpropagation approach. Buildings.

[CR51] Yang, C. (2020). Waterfront recreational landscape planning and ecological protection based on Cloud computing and neural network evaluation, *Data Processing Techniques and Applications for Cyber-Physical Systems* (DPTA 2019), 1789–1798.

[CR52] Ye, Y., Zhao, X., & Hu, Y. (2018). Cultivated land quality evaluation in the Pearl River Delta based on GA-BP neural network. *Journal of Ecology and Environment, 27*(5), 964. http://www.cqvip.com/qk/97636c/20185/675296355.html

[CR53] Zeng L, Hang J, Wang X, Shao M (2022). Influence of urban spatial and socioeconomic parameters on PM2. 5 at subdistrict level: A land use regression study in Shenzhen China. Journal of Environmental Sciences.

[CR54] Zhang Y, Cheng Y, Jiang Y (2019). Research on ecological suitability of public green space in compact cities based on GIS and BP neural network. Journal of Cleaner Production.

[CR55] Zhang, X., Xu, D., & Wang, Z. (2021). Optimizing the spatial layout of afforestation to realize the maximum benefit of water resources in arid regions: A case study of Alxa, China. *Journal of Cleaner Production, 320*, Article ID 10.1016/j.jclepro.2021.128827

[CR56] Zhang X, Duanyang Xu, Wang Z (2021). Optimizing the spatial layout of afforestation to realize the maximum benefit of water resources in arid regions: A case study of Alxa. China. Journal of Cleaner Production.

[CR57] Zhang, H., Feng, C.C., Guo Y.P. (2022). Analysis of the evolution of the spatial pattern and driving factors of the “Three Lives” in urban fringe districts: A case study of Chaoyang District, Beijing, China. Acta Scientiarum Naturalium Universitatis Pekinensis, Vol. 59, No. 3. Doi: 10.13209/j.0479-8023.2023.008

[CR58] Zheng, S., Sun, H., Lou, K., Dong, Q., (2017). The comprehensive evaluation of wetland road ecological landscape based on BP neural network. *Chinese and foreign roads, 2*, 309-314

[CR59] Zou L, Li Y, Li J (2021). Research on urban park landscape quality evaluation based on convolutional neural network. Journal of Cleaner Production.

